# Confidence intervals estimator of the kinetic parameters: do its reliability depend on the assembling method of the oxygen uptakes?

**DOI:** 10.1007/s00421-024-05629-6

**Published:** 2024-10-17

**Authors:** Maria Pia Francescato, Valentina Cettolo

**Affiliations:** Department of Medicine, University of Udine, P.le Kolbe 4, 33100 Udine, Italy

**Keywords:** Fitting window, Phase II, Confidence limits, Stacking procedure, Coverage of the confidence interval

## Abstract

Gas exchange data acquired repeatedly under the same exercise conditions are assembled together to improve the kinetic parameters of breath-by-breath oxygen uptake. The latter are provided by the non-linear regression procedure, together with the corresponding estimators of the width of the Confidence Intervals (i.e., the Asymptotic Standard Errors; ASEs). We tested, for two different assembling procedures, whether the range of values identified by the ASE actually correspond to the 95% Confidence Interval. Ten O_2_ uptake responses were acquired on 10 healthy volunteers performing a square-wave moderate-intensity exercise. Kinetic parameters were estimated running the non-linear regression with a mono-exponential model on an increasingly greater number of responses (Nr, from 1 to 10), assembled together using the “stacking” and the “1-s-bins” procedures. Kinetic values obtained assembling together the 10 repetitions were assumed as “true” values. The time constant was not affected by Nr or by the assembling procedure (ANOVA; *p*>0.54 and *p*>0.16, respectively). The corresponding ASE decreased according to Nr (ANOVA; *p*=0.000), being significantly smaller for the “1-s-bins” procedure compared to the “stacking” one (ANOVA; *p*<0.001). Excluding 20s at the start of the fitting window, the range of values identified with the ASE provided by the “1-s-bins” and the “stacking” procedures included the “true” value in 85% and in 95% of cases, respectively. The “stacking” procedure should be preferred since it yielded ASEs for the time constant that provided a range of values satisfying the statistical meaning of the width of the Confidence Intervals, at the given degree of probability.

## Introduction

At the onset of a square-wave constant-load exercise, after an initial short phase, oxygen uptake at the mouth ($\dot{V}{\text{O}}_{2}$) approaches a new steady state following a time course deemed to be mono-exponential. The parameters describing the $\dot{V}{\text{O}}_{2}$ kinetics, namely the time constant ($\tau$) and the time delay (Td), are affected by an uncertainty that arises mainly from the breath-by-breath fluctuations in gas exchange linked to the inherent irregularities in breathing (Lamarra et al. 1987), and are usually estimated applying the non-linear regression technique.

Similar to any other measurement method, the estimation procedure can be characterized by its “accuracy”, in turn, split according to the International Standard ISO-5725-1 (2023) in: (1) “trueness”, that refers to the closeness between the measured value and the true or accepted reference value, and (2) “precision”, that refers to the closeness of agreement between test results. Commonly, “precision” is expressed in terms of “imprecision”, and can be computed as Standard Deviation, Standard Error, or Variance, etc.

When estimating $\tau$ and Td for a $\dot{V}{\text{O}}_{2}$ kinetics, their “imprecision” can be evaluated by means of the Asymptotic (or Approximate) Standard Error (ASE) provided by the non-linear regression procedure along with the corresponding kinetic parameter. In addition, a range of values can be defined that is included within the following extremes:

1 $${\text{estimated value }} \pm {\text{ t}}_{df} (\alpha ) \, \times {\text{ ASE}}$$

where t_*df*_(α) is the *t* value of the Student’s *t*-distribution for the given two-tails α probability (usually 95%) with *df* degrees of freedom, in turn resulting from the number of samples considered minus the number of estimated parameters. The range of values contained within the above extremes satisfies the statistical definition of “Confidence Interval”, at the α probability level (CI_95_ for α = 95%), when it includes the true or accepted reference (“true”) value in the same percentage of cases. This percentage can be evaluated by the “Coverage of the Confidence Interval” (“coverage”), and, as soon as this is close to α, the range of values included within the extremes obtained with Expression 1 is appropriate to describe the overall “accuracy” of the measurement method, according to the given statistical probability.

Applying a data treatment on the original data before running the non-linear regression procedure makes the range of values included within the above extremes no longer satisfying the definition of “Confidence Interval”. Indeed, previous simulations showed that the “1-s-bins” procedure, i.e., an oversampling data treatment (“cloning”) that replicated the same amount of information on a greater number of values, made the “coverage” falling to about 70% instead of the statistically expected 95% (Francescato et al. 2014a, b).

Commonly, in order to enhance the estimate of the oxygen uptake kinetic parameters, each of the recruited volunteers performs more repetitions of the same square-wave exercise, and all his/her data are then assembled together. Contrasting conclusions were drawn by comparing different methods to assemble the repeated transitions. Indeed, Francescato et al. (2014b), studying the assembling of 10 synthetic repetitions, found that the theoretical definition of Confidence Intervals at 95% probability level was better approximated using the ASE values of the “stacking” procedure (“coverage” >94% of cases), that was thus endorsed, although lower ASE values were found with the “1-s-bins” procedure. Conversely, Keir et al. (2014), who investigated the assembling of 4 experimentally repeated transitions, on the basis of the lower ASE values only, concluded that “The goodness of fit was the highest and confidence in parameter estimation of τ $\dot{V}{\text{O}}_{2}$ p the greatest ... when the non-linear regression model was applied following linear interpolation of individual trials and ensemble averaging”.

The “true” values are required for the assessment of the “coverage”, but they are not known in an experimental setup. Nevertheless, the value estimated from a reasonably great number of assembled $\dot{V}{\text{O}}_{2}$ time series, acquired during the same repeated square-wave exercise, can be considered a robust value to be assumed as “surrogate of the true” one. In a previous experimentation by our workgroup (Francescato and Cettolo 2024), volunteers performed 10 repetitions of the same square-wave moderate-intensity exercise in order to investigate the effects of different time periods excluded at the start of the fitting window. Assuming that the kinetic values obtained assembling all the 10 repetitions performed by the same volunteer could be taken as his/her “surrogates of the true” kinetic parameters, it will be possible to estimate the “coverage” even in experimentally acquired data.

The present work was carried out to test, on experimentally acquired $\dot{V}{\text{O}}_{2}$ data, the effects of two different assembling procedures, i.e., the “stacking” and the “1-s-bins” procedures. In particular, we wanted to assess whether the statistical definition of “Confidence Interval” is satisfied by the range of values obtained on the basis of the estimated parameter and the corresponding ASE (i.e., applying Expression 1), and if the time period excluded at the start of the fitting window has any effect on the “coverage”.

## Methods

### Experimental protocol

The original data are those of a previous experimentation (Francescato and Cettolo 2024); however, according to the purpose of the present investigation, a different data analysis was performed.

Five females and 5 males (*n* = 10), all healthy and moderately active, with mean (± SD) age, stature and body mass of 24.6 ± 3.6 years, 1.73 ± 0.09 m and 73.5 ± 15.1 kg, volunteered to be subjects. Experimental protocol, design, and methods were approved by the Institutional Review Board of the Department of Medicine of the University of Udine (Italy) (#07/2020_IRB issued on March 5th 2020) and conformed to the standards set by the Declaration of Helsinki, except for registration in a database. Volunteers were thoroughly informed about the nature, purpose, and possible risks of the investigation and, thereafter, gave written informed consent to their participation.

In brief, each volunteer repeated the experimental session 5 times at least one day apart. On each experimental session, volunteers performed twice the same 6-min square-wave moderate-intensity exercise bout while continuously pedaling as close as possible to 60 rpm on the ergometer (Corival; Lode B.V., the Netherlands); mechanical power was set to 1.0 W∙kg^−1^ of body mass when Body Mass Index (BMI) was less than 25.0 kg∙m^−2^, whereas for a greater BMI, mechanical power was reduced to 0.95 W∙kg^−1^. The two exercise bouts were preceded by at least 5 min pedaling at 10 W and were separated by no less than 10 min. In agreement with previous experimentations from our workgroup (Francescato and Cettolo 2019; Francescato et al. 2004), the power for the moderate-intensity exercise was chosen according to body mass since we believed that the exercise intensity relative to a precise physiological threshold was not strictly necessary; moreover, this choice allowed avoiding a further visit of each participant to the physiology laboratory for the assessment of lactate threshold or gas exchange threshold, facilitating the recruitment of volunteers for an already demanding experimental protocol.

Respiratory gas collection at the mouth was performed throughout all experimental sessions. The metabolic unit (Metalyzer 3B, Cortex GmbH, Liepzig, Germany) automatically controlled the timings of the protocol and acquired continuously mechanical power, pedaling frequency, heart rate, flow, as well as O_2_ and CO_2_ fractions in inspired and expired air. The analyzers were calibrated according to the procedures indicated by the manufacturer. Breath-by-breath gas exchange was calculated by means of the “Expiration-only” algorithm using the acquired flow and gas fraction traces; details of the computations were described previously (Francescato and Cettolo 2024, 2019).

All the original data as well as the used gas exchange calculation software are available from the corresponding author upon request.

### Data treatment and statistics

All data were analyzed using the *R* environment (R Core Team 2020).

For each subject and each experimental session, all the obtained oxygen uptake time series were split at *t* = 16 min, and the times were shifted setting the start of the two moderate-intensity exercise bouts at *t* = 0. As a result, a total of 10 distinct $\dot{V}{\text{O}}_{2}$ time series were obtained for each volunteer.

**Fig. 1:**
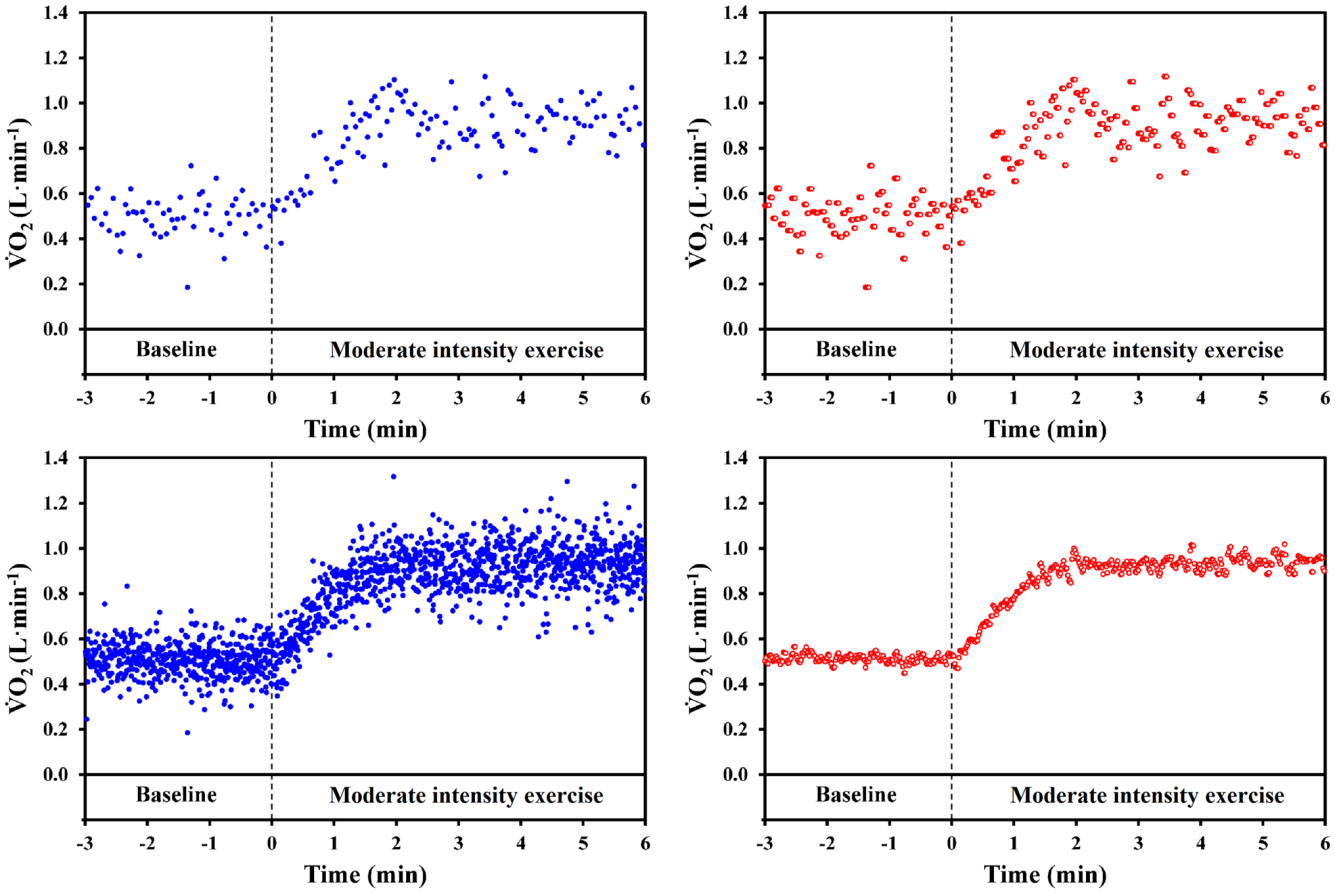
Oxygen uptake data of one volunteer during the first bout of the first experimental session (upper panels) and for all the 10 repetitions assembled together (lower panels). Left panels illustrate the results obtained from the assembling by means of the “stacking” procedure, whereas the right panels illustrate the results of the “1-s-bins” assembling procedure. One repetition included an initial baseline period lasting 3 min while pedaling at 10 W, and a bout of moderate-intensity exercise (55 W for this volunteer) lasting 6 min. Vertical dashed lines correspond to the start of the exercise bout (t = 0 s)

The “1-s-bins” procedure was applied on all the time series to obtain evenly spaced values at 1-s time intervals (starting from *t* = 0 s), where the new time points were paired with a clone (i.e., a copy) of the $\dot{V}{\text{O}}_{2}$ value of the closest native time point, allowing obtaining 10 distinct uniformly spaced time series for each volunteer (Fig. 1, upper panels).

Following the performance order, an increasingly greater number (Nr) of $\dot{V}{\text{O}}_{2}$ time series were assembled together for each volunteer, assuming the start of the square-wave exercise as *t* = 0 s. Two assembling procedures were used: (a) the “stacking” procedure, where the native data pertaining to the distinct time series were simply stacked up to the already included data, and (b) the “1-s-bins” procedure, where the evenly spaced distinct time series were averaged over an increasing number of time series (Bringard et al. 2014; Francescato et al. 2014b). For each volunteer and both assembling procedures, 10 assembled time series were obtained, i.e., taking the first repetition alone (Nr = 1), assembling the first two (Nr = 2), assembling the first three (Nr = 3), the first four (Nr = 4), and so on until Nr = 10 (Fig. 1, lower panels).

The kinetic parameters of $\dot{V}{\text{O}}_{2}$ during the square-wave exercise transition were estimated for all the distinct and all the assembled time series by non-linear regression, using the *nls.lm* routine, without cleaning any outlier, and evaluating the fit by means of the chi-square (χ^2^) value and its statistical significance.

The following mono-exponential model was used in all cases:

2 $$\dot{V}{\text{O}}_{2}\left( {\rm{t}} \right) = {{\rm{A}}_{\rm{b}}} + \Delta {\rm{A}}\left( {1 - {{\rm{e}}^{ - \frac{{{\rm{t}} - {\rm{Td}}}}{\tau }}}} \right){\text{ }}{\text{ }}{\text{ for t} \ge {\rm{Td}}}$$

The starting values for the time constant (τ) and the time delay (Td) were set to 25 s and 0 s, respectively; baseline signal (A_b_) was set to the mean of all the data pertaining to the 3 minutes just before t = 0 s; signal change (ΔA) was set to the difference between the corresponding steady-state signal (mean of all the data pertaining to the 3 min just before t = 360 s) and the baseline one.

The non-linear regression procedure was run 41 times (always applying Eq. 2) on all the $\dot{V}{\text{O}}_{2}$ time series, excluding each time a 1-s progressively longer time period (ΔTr) from the fitting window, starting from t = 0 s (i.e., ΔTr ∈ [0 s, 40 s]), thus yielding for each ΔTr the estimated values for τ, Td, and ΔA, and their Asymptotic Standard Errors (ASE_τ_, ASE_Td_, and ASE_ΔA_, respectively). The subsequent analyses were mainly focused on the results obtained with ΔTr = 0 s or ΔTr = 20 s.

The behavior of the ASE_τ_ (or of the ASE_Td_) values, with the same volunteer and same algorithm, was evaluated by non-linear regression as a function of the number of assembled repetitions (Nr, ranging from 1 to 10) according to the following equation:

3 $${\text{ASE}} = { }\frac{k}{{\sqrt {{\text{Nr}}} }}$$

where *k* represents the ASE value extrapolated for Nr = 1.

The Analysis of Variance for repeated measures (2 × 10 ANOVA) was used to detect the significant differences for the kinetic parameters and their corresponding ASE values, with the following Within-Subjects effects: between the two data treatments (*Treatment* effect) and among the number of repetitions, both distinct (*Repetition* effect) and assembled (*Repeats* effect). Helmert post hoc contrast was used to assess the specific differences within the *Repetition* or *Repeats* effect.

Finally, the “Coverage of the Confidence Interval” was calculated for τ, and Td, as the percentage responses where the range of values calculated by Expression 1 (i.e., estimated value ± t_*df*_(α) ∙ ASE, for two-tails α probability = 95%) included the “surrogate of the true” value. The latter was assumed to be the corresponding kinetic value estimated after the assembling, with the same procedure, of all the 10 repetitions. Of note, theoretically, for the two-tails α probability = 95%, the ASE values allow getting the width of the Confidence Interval, satisfying its statistical meaning, only if the “coverage” results ≅95%.

Significance level was set at *p* < 0.05. Summarized values are reported as means ± SD.

## Results

Table 1 summarizes the F ratios, and corresponding *p* values, yielded by the Analysis of Variance for repeated measures performed on the temporal parameters and the corresponding ASEs obtained for the 10 distinct kinetics, with *Treatment* and *Repetition* as Within-Subjects effects. The temporal parameters were obtained by including in the fitting window all the $\dot{V}{\text{O}}_{2}$ data (ΔTr = 0 s) or by excluding the data pertaining to the first 20 s (ΔTr = 20 s); the average values according to the treatment are reported in the last two columns.

**Table 1:** Statistical results of the Analysis of Variance for repeated measures applied on the temporal parameters of the $\dot{V}{\text{O}}_{2}$ kinetics obtained for the 10 distinct repetitions of each volunteer

		Effect				“stacking”	“1-s-bins”
		Repetition		Treatment		Mean ± SD	Mean ± SD
		F ratio	*p*	F ratio	*p*	(s)	(s)
ΔTr = 0 s	τ	0.78	0.640	14.18	**0.004**	33.2±2.6	31.9±1.9
	Td	0.48	0.885	10.15	**0.011**	13.6±1.8	14.6±1.6
	ASE_τ_	0.71	0.701	52.70	**0.000**	7.7±0.7	4.2±0.3
	ASE_Td_	0.69	0.714	53.77	**0.000**	4.7±0.4	2.6±0.2
ΔTr = 20 s	τ	0.93	0.505	0.54	0.481	28.8±2.1	29.2±1.8
	Td	0.21	0.992	4.25	0.069	18.1±1.1	17.4±0.9
	ASE_τ_	0.58	0.813	41.76	**0.000**	8.0±0.7	4.5±0.3
	ASE_Td_	0.53	0.847	37.27	**0.000**	5.9±0.6	3.2±0.3

F ratios and corresponding *p* values obtained from the Analysis of Variance for repeated measures are summarized for the time constant and time delay of the $\dot{V}{\text{O}}_{2}$ kinetics, and corresponding ASE values. The analysis was performed on the parameters obtained for the 10 volunteers, running the non-linear regression including all the $\dot{V}{\text{O}}_{2}$ data (ΔTr = 0 s), or excluding the data pertaining to the first 20 s (ΔTr = 20 s) from the start of the transient. *Repetition* (*n* = 10) and *Treatment* (*n* = 2) were used as Within-Subjects effects. Bold characters highlight the statistically significant effects (*p* < 0.05). The last two columns summarize the average values according to the treatment, the SDs representing the variability among repetitions

Since no significant *Repetition* effect was observed, an increasingly greater number of repetitions were assembled together, following the performance order.

**Fig. 2:**
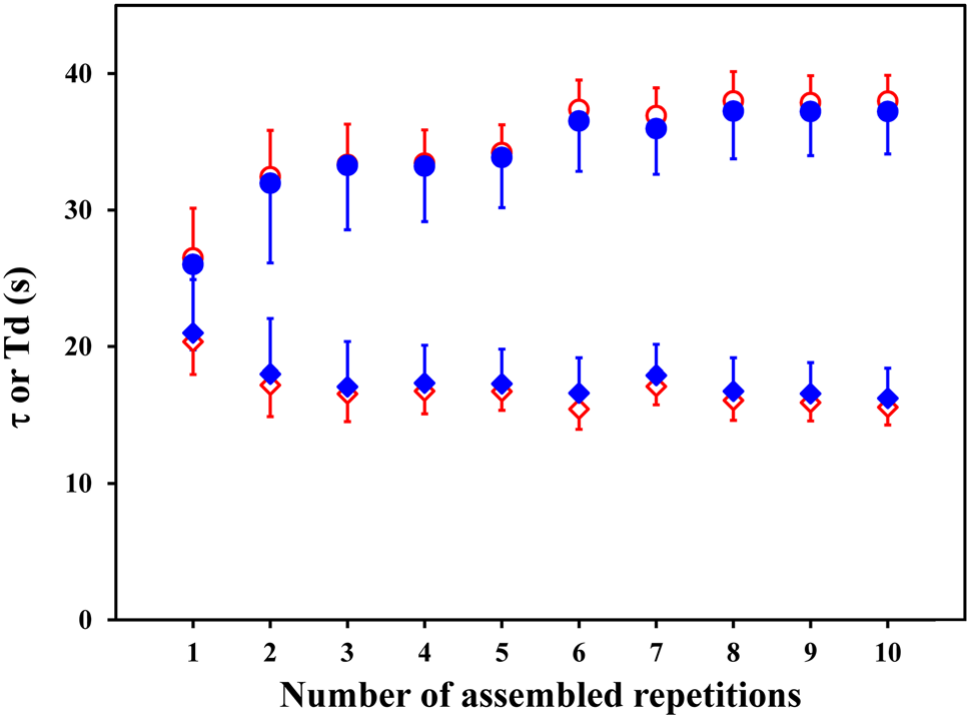
The behaviors of the time constant (τ; dots) and of the time delay (Td; diamonds) are illustrated as a function of the number of assembled repetitions (Nr) for the same volunteer as in Fig. 1. The parameters obtained for the $\dot{V}{\text{O}}_{2}$ data assembled by the “stacking” procedure (full blue symbols) or by the “1-s-bins” procedure (open red symbols) are shown. Values were estimated excluding the $\dot{V}{\text{O}}_{2}$ data pertaining to the first 20 s (ΔTr = 20 s) from the start of the transient. Vertical bars are the corresponding Asymptotic Standard Errors. It can be noted that the assembling more repetitions attenuates fluctuations of parameters within the various repetitions (as clearly shown by the values of the first repetitions), leading to apparently stable values. The number of repetitions required for each volunteer, however, cannot be set *a priori* and has to be chosen as a compromise between the individual response and the purpose of the measurement

Figure 2 illustrates, for one volunteer, the behaviors of the time constant (τ) and of the time delay (Td) obtained with ΔTr = 20 s, as a function of the number of assembled repetitions, using the “stacking” or the “1-s-bins” procedure. It can be noted that, independent of the used assembling procedure, the ASE values (the vertical bars) decrease with increasing Nr for both τ and Td values.

Table 2 summarizes the F ratios, and corresponding *p* values, yielded by the Analysis of Variance for repeated measures performed on the temporal parameters and the corresponding ASEs obtained for the kinetics resulting from an increasingly greater number of assembled repetitions, with *Treatment* and *Repeats* as Within-Subjects effects. Results obtained including in the fitting window all the $\dot{V}{\text{O}}_{2}$ data (ΔTr = 0 s), or excluding the first 20 s (ΔTr = 20 s), are reported. The τ values, as well as the Td values, were not affected by the applied assembling procedure (*Treatment* effect), and by the number of assembled repetitions (*Repeats* effect); the grand averages of τ amounted to 33.1 ± 7.5 s and 29.3 ± 8.4 s for ΔTr = 0 s and ΔTr = 20 s, respectively. A *Repeats* effect was detected for both the ASE_τ_ and the ASE_Td_ values, independent of the assembling procedure used and of the fitting window, their decrease being statistically linked to the increase of Nr (Post-hoc Helmert contrast; F-ratio > 9.5, *p* < 0.013). Significantly smaller ASE_τ_ and ASE_Td_ values were obtained for the “1-s-bins” procedure compared to the “stacking” procedure (*Treatment* effect).

**Table 2:** Statistical results of the Analysis of Variance for repeated measures applied on the temporal parameters of the $\dot{V}{\text{O}}_{2}$ kinetics obtained for each volunteer by assembling together an increasing number of his/her $\dot{V}{\text{O}}_{2}$ responses, following the performance order

		Effect			
		*Repeats (Nr)*		*Treatment*	
		F ratio	*p*	F ratio	*p*
ΔTr = 0 s	τ	0.88	0.545	2.26	0.167
	Td	0.75	0.661	1.66	0.230
	ASE_τ_	22.81	**0.000**	40.01	**0.000**
	ASE_Td_	21.27	**0.000**	39.39	**0.000**
ΔTr = 20 s	τ	0.79	0.626	0.23	0.598
	Td	0.34	0.960	2.58	0.142
	ASE_τ_	20.88	**0.000**	27.66	**0.001**
	ASE_Td_	17.69	**0.000**	22.17	**0.001**

F ratios and corresponding p values obtained from the Analysis of Variance for repeated measures are summarized for the time constant and the time delay of the $\dot{V}{\text{O}}_{2}$ kinetics, along with the corresponding ASE values. The analysis was performed on the parameters obtained for the 10 volunteers, running the non-linear regression either including all the $\dot{V}{\text{O}}_{2}$ data (ΔTr = 0 s), or excluding the data pertaining to the first 20 s (ΔTr = 20 s) from the start of the transient. *Repeats* (*n* = 10) and *Treatment* (*n* = 2) were used as Within-Subjects effects. Bold characters highlight the statistically significant effects (*p* < 0.05)

**Fig. 3:**
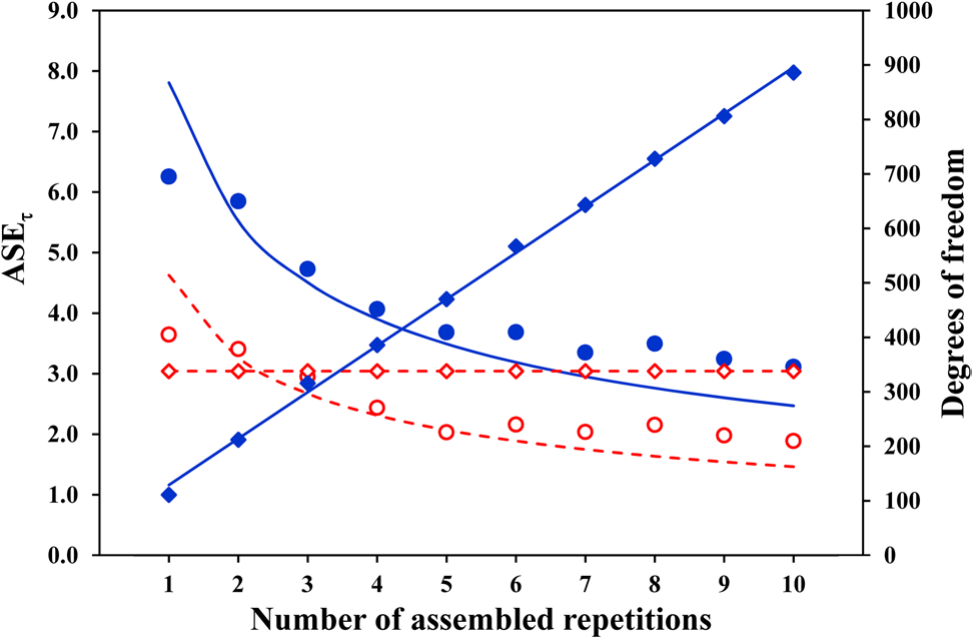
Asymptotic Standard Errors and degrees of freedom, obtained by estimating the time constants, are illustrated as a function of the number of assembled repetitions (Nr) for the same volunteer as of Fig. 1. The ASE_τ_ values yielded by both the “stacking” procedure (full blue dots and continuous line) and the “1-s-bins” procedure (open red dots and dashed line) are shown; all the values were obtained excluding from the fitting window the $\dot{V}{\text{O}}_{2}$ data pertaining to the first 20 s from the start of the transient (ΔTr = 20 s). The two curves of the ASE values are drawn using the parameters estimated by Eq. 3. The corresponding degrees of freedom increase quite linearly for the “stacking” procedure (full blue diamonds), whereas they remain constant for the “1-s-bins” procedure (open red diamonds)

The effect of the assembling of more repetitions (Nr) on the ASE_τ_ values, as well as on the degrees of freedom, is illustrated in Fig. 3 for one volunteer; data were obtained applying both assembling procedures with ΔTr = 20 s. The ASE_τ_ values decrease with increasing Nr for both assembling procedures, whereas the degrees of freedom increase quite linearly for the “stacking” procedure, but remain constant for the “1-s-bins” procedure.

For all the volunteers, the decrease of the ASE_τ_ and ASE_Td_ values with increasing Nr was approximated by Eq. 3 for both assembling procedures and both fitting windows (χ^2^ < 2.40; *p* < 0.016 for all).

**Fig. 4:**
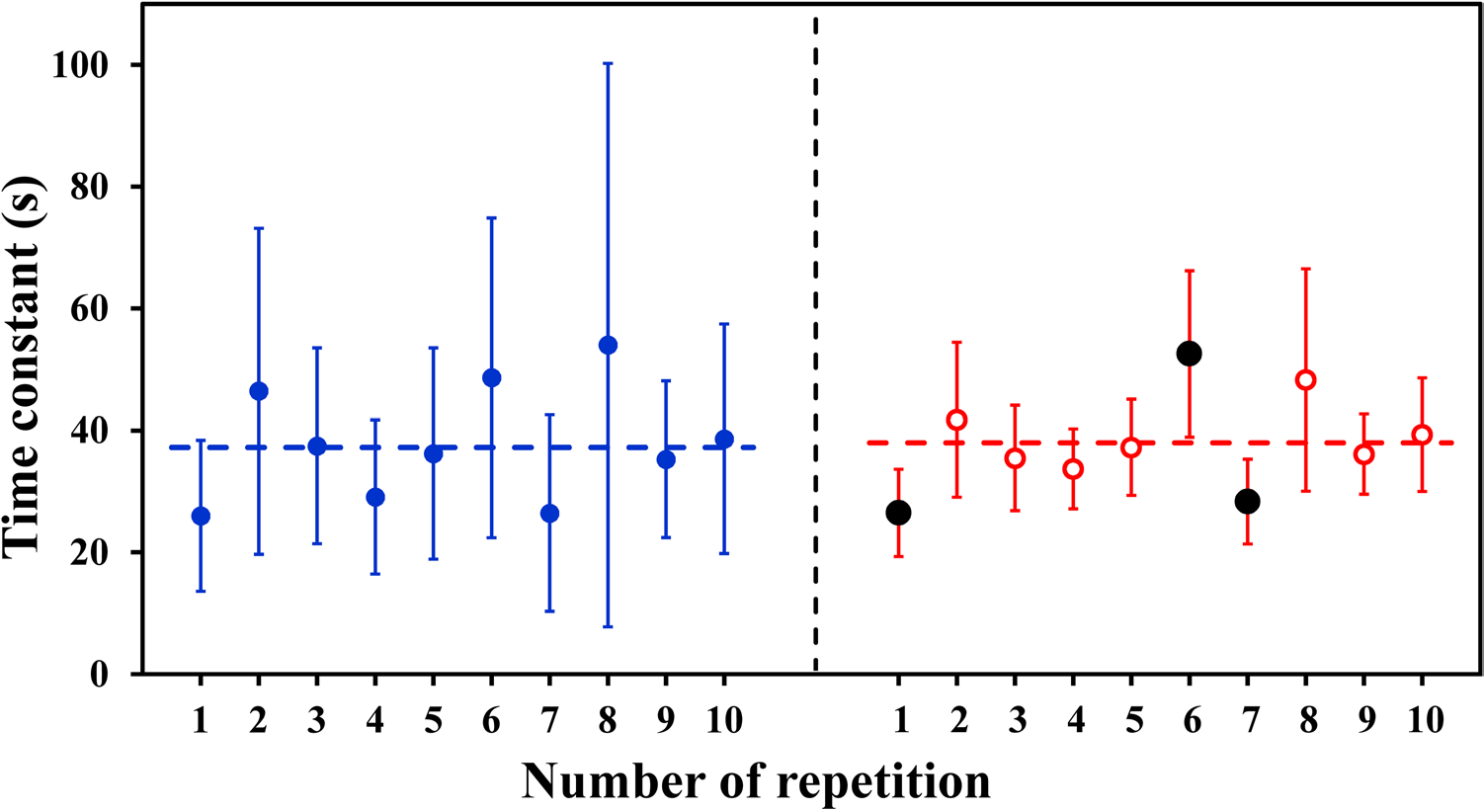
Example on how the “coverage” of the Confidence Interval was calculated for the same volunteer as in Fig. 1. The τ values are illustrated for each distinct repetition for both the “stacking” procedure (full blue dots) and the “1-s-bins” procedure (open red dots); all values were obtained excluding from the fitting window the $\dot{V}{\text{O}}_{2}$ data pertaining to the first 20 s from the start of the transient (ΔTr = 20 s). Horizontal lines represent the corresponding τ values obtained for the 10 repetitions assembled together, assumed as “surrogate of the true” value. Vertical bars are the specific range of values of each estimated time constant, obtained as τ ± t_*df*_(α)∙ASE_τ_ (Expression 1). It can be noted that, for the “stacking” procedure, the vertical bars include the “surrogate of the true” value in all cases, whereas for the “1-s-bins” procedure, they fail in including the “surrogate of the true” value in 3 cases (repetitions *n*. 1, 6 and 7, highlighted by a black full symbol)

The method used to calculate the “Coverage of the Confidence Interval” is illustrated in Fig. 4 for the time constant of one volunteer. The τ estimated for the distinct repetitions is shown for both data treatments as a function of the number of repetition, with the vertical bars representing the extensions of the range of values calculated by Expression 1 for a two-tails α probability of 95%; the two horizontal lines correspond to the time constants estimated for the 10 repetitions of that volunteer assembled together using the corresponding procedure, and assumed to represent the “surrogate of the true” time constant of the kinetics. The figure highlights that the range of values calculated by Expression 1 for the “1-s-bins” procedure fails in including the assumed “surrogate of the true” value in 3 cases (repetitions n. 1, 6 and 7). Conversely, the range of values obtained for the “stacking” procedure includes the assumed “surrogate of the true” value in all the repetitions.

**Fig. 5:**
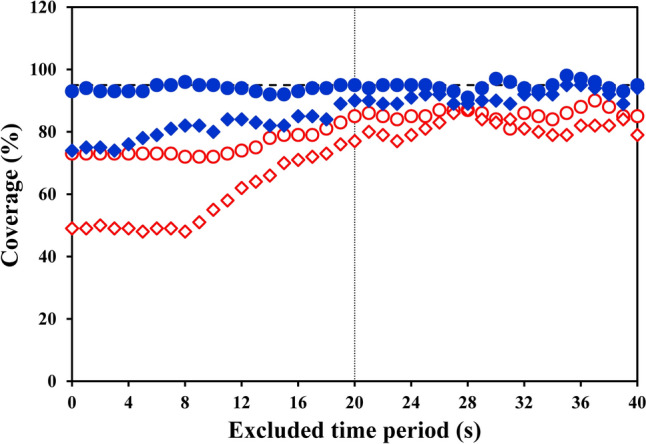
“Coverage of the Confidence Intervals” for τ (dots) and Td (diamonds) as a function of the time period excluded from the fitting window (ΔTr) are illustrated for both the “stacking” (blue full symbols) and the “1-s-bins” procedure (red open symbols). The “coverage” for the τ values obtained with the “stacking” procedure is close to 95% for all the excluded time periods, the corresponding Td reaching a quite stable “coverage” (near 90%) for ΔTr > 20 s. The “1-s-bins” procedure yielded a “coverage” for both τ and Td always lower than the “stacking” procedure, reaching at most 87% for τ. Dashed horizontal line indicates 95% “coverage” of the Confidence Interval; thin vertical line corresponds to ΔTr = 20 s

Fig. 5 illustrates, for both assembling procedures, the “Coverage of the Confidence Interval” for τ and Td calculated over all the volunteers and repetitions (10 × 10 = 100 cases) as a function of the time period excluded from the fitting window (ΔTr). The “stacking” procedure yielded a “coverage” for τ close to 95% (the theoretical statistical value to be reached) independent of ΔTr, whereas the “coverage” reached at most 87% for τ obtained by means of the “1-s-bins” procedure.

## Discussion

To the best of our knowledge, this is the first paper trying to evaluate, in an experimental setup, the “Coverage of the Confidence Interval” of the $\dot{V}{\text{O}}_{2}$ kinetic parameters. The assessment of the “coverage” was based on: (1) the Asymptotic Standard Errors provided by the non-linear regression procedure, and (2) the “surrogate of the true” value, obtained from the ten $\dot{V}{\text{O}}_{2}$ responses of the same volunteer assembled together, assumed to reliably represent the “true” value. Results highlight that the ASE might not be appropriate to identify the range of values that satisfy the statistical definition of “Confidence Interval” when pre-processing of the data affects the information contained in the samples supplied to the non-linear regression.

### The effects of an increasing number of assembled repetitions

This experimentation confirms that the estimated kinetic parameters were not affected by the number of repeated and assembled repetitions (Nr). Conversely, results provide proof that the “precision” of the oxygen uptake kinetic parameters is improved as a function of Nr, independent of the degrees of freedom. Indeed, for the same volunteer, a statistically significant relationship with the reciprocal of the square root of Nr was detected for the ASE values when the number of assembled repetitions was increased. This behavior was observed for both the investigated assembling procedures and for both the investigated fitting windows (i.e., ΔTr = 0 s and ΔTr = 20 s).

A few readers will consider it logical that ASE reduces as a function of the inverse of the square root of Nr because more samples are supplied to the non-linear fitting. This reasoning might hold true for the “stacking” procedure, but Fig. 3 shows that ASE_τ_ decreased as a function of Nr also for the “1-s-bins” procedure, where the number of samples supplied to the non-linear fitting remained constant, independent of Nr. The decrease of the ASE_τ_ for the “1-s-bins” procedure might be explained by the fact that this procedure produces “mean physiological responses that are smoothed by a filter whose time constant is the mean breath duration (typically 3–4 s for these studies).” (Lamarra et al. 1987). Notably, the higher the number of averaged repetitions, the more efficient will become the smoothing, thus leading to a reduction of the ASE despite the number of samples supplied to the non-linear fitting remain the same.

**Fig. 6:**
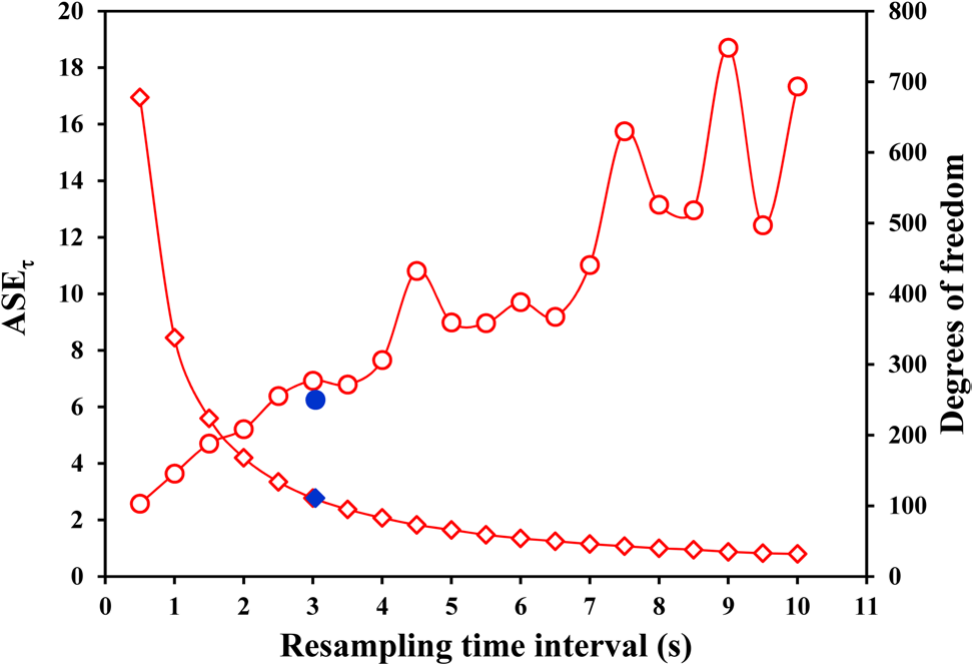
Effect of the resampling time interval on the ASE_τ_ values (open red dots) and the corresponding degrees of freedom (open red diamonds) obtained by means of the non-linear fitting for the data of the first repetition of the same volunteer as of Fig. 1, excluding from the fitting window the $\dot{V}{\text{O}}_{2}$ data pertaining to the first 20 s from the start of the transient (ΔTr = 20 s). The ASE_τ_ value increased, and the corresponding degrees of freedom decreased, with increasing the resampling time interval. For comparison, the ASE_τ_ value obtained with the “stacking” procedure (full blue dot), as well as the corresponding degrees of freedom (full blue diamond), is shown in correspondence with the native average time interval

Independent of the number of assembled repetitions, a drawback of the filter applied when using the “1-s-bins” procedure is that the fluctuations of the native data are reduced, leading to artificially smaller ASE values compared to those obtained with the “stacking” procedure. The “filter” has different effects on the Asymptotic Standard Error according to the resampling time interval of the native data, as illustrated in Fig. 6 for the ASE_τ_ values obtained for the first repetition alone of one volunteer. The figure highlights that the ASE_τ_ value becomes smaller as the resampling time interval becomes shorter; moreover, for a resampling time interval close to the average time interval of the native data, the ASE_τ_ value is similar to that obtained applying the “stacking” procedure.

### The Coverage of the Confidence Interval

It can be questioned which one of the ASE_τ_ values (that resulting from the “1-s-bins” or from the “stacking” procedure) is appropriate to evaluate the width of the Confidence Interval at the given degree of statistical significance.

A threshold of 95% of “coverage” has to be set to be complementary with the statistical significance level set at *p* < 0.05. The ASE_τ_ values obtained using the “stacking” procedure included the assumed “surrogate of the true” value in 95% of cases (for the whole range of time periods excluded from the start of the transient). Conversely, although appealing at first because smaller, the ASE_τ_ values obtained with the “1-s-bins” procedure allowed inclusion of the assumed “surrogate of the true” value in at most 87% of cases (Fig. 5). This result is in line with the results published previously on synthetic data (Francescato et al. 2014a, b). Indeed, when a resampling procedure with a time interval shorter than the average breath duration is applied (“cloning”), the overall information contained in the data remains the same, whereas the number of samples supplied to the non-linear fitting increases; the obtained ASE values are reduced because the square root of the degrees of freedom is part of the denominator of the ASE equation (Press et al. 2007). Under these conditions, the ASE values have lost their original statistical meaning, leading to a “coverage” lower than 95%.

The “coverage” obtained in the present investigation was somewhat higher than that resulting from a previous simulation (Francescato et al. 2014b), in particular when the “1-s-bins” procedure was applied. In the present investigation, however, we evaluated the 10 distinct repetitions of each volunteer assuming as “surrogate of the true” value that estimated assembling all the 10 repetitions together; thus, the “true” value and the values to be compared are not fully independent.

### Risks of erroneous interpretations from erroneously estimated Confidence Intervals

As reported above, when native data are not manipulated before the assembling of repeated transitions, the ASE_τ_ provided by the non-linear regression procedure allows identifying a range of values that satisfy the statistical definition of Confidence Interval of the estimated parameter, with lower ASEs resulting in narrower CIs. In turn, the latter are frequently used to evaluate the goodness of the individual fits although in too many cases, this information is not provided in the papers, not even as average values (e.g., Keir et al. 2016). It should be emphasized, however, that universally accepted thresholds of the CI to define the “goodness” of fit are not available, either expressed in absolute and in relative terms.

The comparison of the goodness of the reported kinetic data might be hampered when the CIs are reported after application of different manipulations of the native data (e.g., Love et al. 2023; Spencer et al. 2012). The same difficulty might be encountered within the same experimentation when the manipulations concern different physiological signals (e.g., $\dot{V}{\text{O}}_{2}$, NIRS data, cardiac output), acquired on the same volunteer with different time resolutions (e.g., Goulding et al. 2018).

The comparison of different groups of kinetic data (e.g., before/after a treatment, groups of different age) might lead to different interpretations depending on the width of the CIs, where apparently distinct groups (showing narrow CIs due to native data manipulation) might in reality be overlapping when the correct width of the CIs is considered (e.g., Murias et al. 2011).

Finally, it should be good practice to illustrate also the corresponding CIs when relationships between kinetic parameters of different physiological variables are shown. In these cases, however, only the appropriate ASE values will represent the correct statistical variability of each data point. Indeed, different CIs might have led to different conclusions when comparing the time constants of phosphocreatine breakdown and of oxygen uptake (Rossiter et al. 1999), or the relationship between phosphocreatine concentration in muscles and τ of its breakdown (Francescato et al. 2008).

In conclusion, in order to avoid the risk of erroneous interpretations arising from an erroneous assessment of the uncertainty associated with the estimated parameters, the repeats of the same volunteer should be assembled together using the “staking” procedure (e.g., Fontolliet et al. 2021; Taboni et al. 2024).

## Strength and limitations

The “trueness” of the parameters estimated from the 10 distinct repetitions of one volunteer was assessed using as reference value (“surrogate of the true” value) that estimated after the assembling of the same repetitions. A large number of repetitions yielding the kinetic parameters should be evaluated against the “true” value, provided, in turn, by a huge number of different repetitions assembled together. Such a protocol would be very demanding, making it difficult to recruit a sufficient number of volunteers. It is obvious that experimentally acquired data can never reach the number of cases that can be evaluated by means of simulated synthetic data, which, however, can only approximate the reality.

The analysis was limited to the “Expiration-only” algorithm. It cannot be excluded that the same analysis carried out on gas exchange data obtained by means of other calculation algorithms (Cettolo and Francescato 2018), where the fluctuations (i.e., noise) are different, might bring to different results. The inclusion of more algorithms in the analysis performed in the present work was not considered because it would make the paper less manageable and difficult to be read. A similar difficulty for the reading of the paper would arise if more data treatments had to be compared besides the two procedures at stake (i.e., the “stacking” and the “1-s-bins” procedures).

The analysis was performed using a mono-exponential model although two main phases are deemed to occur throughout the transient period (Barstow and Molé 1987; Ferretti 2015; Poole and Jones 2012): the first phase is believed to represent the cardio-dynamic adjustment to the exercise; the second phase (also called primary phase) is assumed to be the image of the O_2_ uptake at the muscle level. The most appropriate model should be a bi-exponential; nevertheless, the more complex is the model, the higher is the number of descriptive variables, making their estimate less robust (Motulsky and Ransnas 1987). Consequently, the number of repeats required to obtain sufficiently robust “surrogates of the true” values should be increased well over ten repeats.

The analyses were mainly focused on the results obtained with ΔTr = 0 s or ΔTr = 20 s. In the first case, results have their meaning since they allow the calculation of O_2_ deficit from the Mean Response Time of  $\dot{V}{\text{O}}_{2}$ at the start of the square-wave exercise (Ferretti et al. 2022). Discarding the data pertaining to the first 20 s from the start of the exercise (the second case) is a commonly used procedure since it is deemed to avoid interferences from the cardio-dynamic phase (Murias et al. 2011).

The group of recruited volunteers was quite homogeneous because they had a rather narrow range of ages and all of them were healthy (Francescato and Cettolo 2024). It cannot be excluded that the recruitment of younger or older volunteers, or suffering from pathological conditions, might lead to different results, which, in turn, might be interpreted in a different way.

Exercise intensity was chosen according to volunteer’s body mass, not relative to an individual physiological threshold during exercise (e.g., gas exchange threshold or lactate threshold). During a constant intensity exercise below the above thresholds, however, following the cardio-dynamic phase and the primary phase, gas exchange remains quite constant and does not show a temporal drift (the so-called slow component). As already reported in our previous paper (Francescato and Cettolo 2024), a non-statistically significant slope was found in the gas exchange data during steady state in all volunteers, suggestive of the absence of a slow component.

The present experimentation was limited to the moderate-intensity exercise domain and no results are shown for heavier exercise intensities. However, it can be expected that the “1-s-bins” interpolation procedure will still “smooth by a filter” the obtained $\dot{V}{\text{O}}_{2}$ responses. We are thus confident that the “stacking” procedure, that does not manipulate the native data, will still provide ASE values that are better estimators of the width of the Confidence Intervals.

## Conclusions

This paper confirms, in experimentally acquired data, that the “precision” of the oxygen uptake kinetic parameters is improved by increasing the number of repeated and assembled transients. Nevertheless, as soon as the same amount of information is replicated on a large number of samples (e.g., using an oversampling procedure), misleading small Asymptotic Standard Errors are obtained. Conversely, the non-linear regression procedure applied on the native data produces ASE_τ_ values resulting in a “coverage” near to the expected one, even when more repetitions of the same transient are assembled together. Consequently, the “stacking” procedure yields ASE_τ_ values that are appropriate estimators of the width of the Confidence Intervals and thus this should be the procedure of choice.

## Data Availability

Data supporting the findings of the present paper as well as the used software are available from the corresponding author upon reasonable request.

## Abbreviations

Ab: Baseline signal
ANOVA: Analysis of variance
ASE: Asymptotic (or Approximate) Standard Error (generic)
BMI: Body mass index
CI_95_: 95% Confidence interval
df: Degrees of freedom
ΔA: Signal change
ΔTr: Time period excluded from the fitting window
Nr: Number of assembled repetitions
SD: Standard deviation
t: Time (generic)
$$\tau$$: Time constant
Td: Time delay
$t_{df} (\alpha )$: Quantile of the Student’s t-distribution for two-sided $\alpha$ probability level with df degrees of freedom
"true": True or accepted reference value according to the International Standard ISO-5725-1 (2023)
$\dot{V}{\text{O}}_{2}$: Oxygen uptake value
